# Which factors are associated with global cognitive impairment in Wilson's disease?

**DOI:** 10.1590/s1980-5764-2016dn1004011

**Published:** 2016

**Authors:** Norberto Anízio Ferreira Frota, Egberto Reis Barbosa, Claudia Sellitto Porto, Leandro Tavares Lucato, Carla Rachel Ono, Carlos Alberto Buchpiguel, Alexandre Aluizio Costa Machado, Paulo Caramelli

**Affiliations:** 1 MD, PhD. School of Medicine, University of Fortaleza, Fortaleza, CE, Brazil. MD, PhD.; 2 Department of Neurology, University of São Paulo School of Medicine, São Paulo, SP, Brazil.; 3 PhD. Department of Neurology, University of São Paulo School of Medicine, São Paulo, SP Brazil.; 4 MD, PhD. Department of Radiology, University of São Paulo School of Medicine, São Paulo, SP, Brazil.; 5 MD, PhD. Department of Internal Medicine, Faculty of Medicine, Federal University of Minas Gerais, MG, Brazil.

**Keywords:** Wilson's disease, cognitive impairment, neuropsychological tests, magnetic resonance imaging, globus pallidus

## Abstract

**Background:**

Patients with Wilson's disease (WD) present cognitive impairment, especially in executive functions. Which other factors might be associated with global cognitive decline in these patients remains unclear.

**Objective:**

To assess which factors are associated with worse performance on a global cognitive test in patients with WD.

**Methods:**

Twenty patients with WD underwent cognitive assessment with the following tests: the Mini-Mental State Examination (MMSE), Dementia Rating Scale (DRS), verbal fluency test, brief cognitive battery, clock drawing test, Frontal Assessment Battery, Stroop test, Wisconsin card sorting test, Hopper test, cubes (WAIS) and the Pfeffer questionnaire. MRI changes were quantified. Patients with poor performance on the DRS were compared to patients with normal performance.

**Results:**

Nine patients had a poor performance on the DRS. This group had lower educational level (9.11±3.58× 12.82±3.06) and a greater number of changes on MRI (9.44±2.74× 6.27±2.45). The presence of hyperintensity in the globus pallidus on MRI was more frequent in this group (66.6% vs 9.0%), with OR=5.38 (95% CI 0.85-33.86).

**Conclusion:**

Global cognitive impairment was prevalent in this sample of patients with WD and was associated with low educational level, number of changes on MRI and MRI hyperintensity in the globus pallidus.

## Abstract (pt)

**Embasamento:**

Pacientes com doença de Wilson (DW) apresentam comprometimento cognitivo, principalmente de funções executivas. Existem dúvidas sobre quais outros fatores poderiam estar associados ao declínio cognitivo global nesses pacientes.

**Objetivo:**

Avaliar quais fatores estão associados ao pior desempenho em teste cognitivo global em pacientes com DW.

**Métodos:**

Vinte pacientes com DW em tratamento regular foram submetidos à avaliação cognitiva com os seguintes testes: Mini-Exame do Estado Mental, escala de demência de Mattis (DRS), fluência verbal, bateria cognitiva breve, desenho do relógio, bacteria de avaliação frontal, Stroop, teste de seleção de cartões de Wisconsin, Hopper, Cubos e ao questionário de Pfeffer. As alterações em RM foram quantificadas. Pacientes com desempenho alterado na DRS foram comparados aos pacientes com desempenho normal.

**Resultados:**

Nove pacientes apresentavam desempenho alterado na DRS. Eles apresentavam menor nivel educacional (9,11±3,58 × 12,82±3,06 anos, respectivamente) e maior quantidade de alterações na RM (9,44±2,74 × 6,27±2,45). A presença de hipersinal no globo pálido na RM foi mais frequente nesse grupo (66,6% × 9,0%), com OR=5,38 (IC 95% 0,85-33,86).

**Conclusão:**

Alterações cognitivas globais foram frequentes nesta amostra de pacientes com DW e se associaram à baixa escolaridade, quantidade de alterações em RM e a hipersinal no globo pálido à RM.

## INTRODUCTION

Wilson's disease (WD) is a rare genetic condition described over 100 years ago, characterized by the accumulation of copper in many organs, including the central nervous system.^1^

The cognitive symptoms present in the first recorded patients were not studied in depth for some time.^2^ The first studies assessing cognitive performance described changes in memory and executive functions in patients with neurological symptoms of WD,^3,4^ raising doubts as to whether the motor symptoms were in fact responsible for the cognitive impairment.^3^ Patients with only hepatic symptoms did not exhibit cognitive abnormalities.^5^

In 2002, a study found that patients with lesions restricted to the basal nuclei displayed cognitive changes compared to healthy controls.^6^ Recently, it has been noted that patients with neurological symptoms but no depressive or anxiety disorders, present cognitive impairment, mainly in executive functions. Furthermore, the number of deficits on cognitive tests was associated with the intensity of brain Magnetic Resonance Imaging (MRI) changes, especially hyperintense signals on T2 images and atrophy.^7^

A study of 12 patients with WD sought to correlate the topographies of hyperintensities on MRI with cognitive profile and suggested that changes in the putamen are related to worse performance on cognitive tests.^8^ However, the small number of patients and the fact that only descriptive analysis of the data was performed limit this finding.

Nevertheless, it is known that the intensity of changes on MRI and the severity of motor symptoms are related to cognitive changes.^7^

Not all patients with WD exhibited deficits on the cognitive tests. Normal overall performance on cognitive tests can be observed in more than 50% of WD patients.^3,7^ It is not clear which factors are related to cognitive symptoms, such as disease duration, medications used or the specific topography of T2-hyperintensity changes.

In the present study, data previously published by us was reevaluated, comparing a group of patients with WD that exhibited deficits on global cognitive tests against cognitively unimpaired WD patients.

## METHODS

The methodology used to select the patients, as well as the cognitive evaluation and neuroimaging protocols, has been described in detail elsewhere.^7^ A brief description is given below.

**Patients.** Twenty patients with WD were selected based on clinical history, physical examination, serum ceruloplasmin levels (<20 mg/dl), 24-h urinary copper excretion (>100 mcg/24 h) and ophthalmologic examination by slit lamp. All patients were followed at a Movement Disorders Clinic from September 2006 to October 2007. The patients were undergoing regular treatment for at least one year, without neurological decline. Patients with severe dysarthria or anarthria that could impair speech comprehension, as well as those with motor disorders which could prevent them from performing written tasks, as well as clinical signs of hepatic encephalopathy, depression/anxiety according to the Goldberg scale, were excluded.^9^ All patients signed a consent form and the study was approved by the institution's Ethics Committee.

**Neurological evaluation.** All patients were evaluated by the lead researcher of the study (NAFF) using the motor symptoms scale (MSS)^7,10^ which assesses 13 items (bradykinesia, rigidity, postural instability, tremor, dystonia, chorea, athetosis, cerebellar disturbances, dysarthria, dysphagia, walking difficulties, psychiatric disturbances and other alterations), each graded from 0 to 3 (absent, slight, moderate and intense). Hence, the total score on the scale ranges from 0 to 39.

**Cognitive evaluation.** All patients were submitted on different days to a two-stage cognitive evaluation, each lasting around 40 minutes.

The first session was applied by the same examining neurologist (NAFF) and included the following tests: Mini-Mental State Examination;^11^ a memory test of figures,^12^ clock drawing;^13^ verbal fluency tests (animals and FAS); CERAD naming test;^14^ Stroop test^15^ and the Frontal Assessment Battery (FAB).^16^ The patients' relatives also answered the Pfeffer Functional Activities Questionnaire.^17^

The second evaluation session was performed by an experienced neuropsychologist (CSP), with a similar duration to that of the first evaluation, involving the following tests: Mattis Dementia Rating Scale (DRS),^18^ Wisconsin Card Sorting Test (WCST) and the Hooper and Cubes tests (subscale of Wechsler intelligence scale).

**DRS performance parameters.** In order to define presence of global cognitive changes, education-adjusted DRS total scores were used, namely: <126 for individuals with 0-4 years of education;<130 for those with 5-12 years; and <136 for subjects with an educational level of ≥13 years. These cut-off scores were based on the rules suggested by Porto et al. in the Brazilian population,^19^ using percentile values ≤10.

**MRI.** Magnetic resonance imaging examinations were performed using a 1.5 Tesla according to a previously published acquisition protocol.^7^ The analyses of the images were performed by an experienced neuroradiologist (LTL), blinded to the clinical and cognitive pictures of the patients, using a scale^7^ for quantitative assessment of the MRI structural changes. On the scale, one point is given in the presence of a hyperintense T2 signal in each of the following structures (maximum 7 points): putamen, globus pallidus, caudate, thalamus, mesencephalon, cerebellum and cerebral white matter. An additional point is given for the presence of a hypointense T2 signal (SE) in the following structures (maximum 6 points): globus pallidus, putamen, caudate, red nucleus, substantia nigra and dentate nucleus of the cerebellum. The presence of a hyperintense T1 signal in the globus pallidus was also attributed one point. In virtue of lesions classically being bilateral and symmetrical, laterality was not taken into account. The last two points of the scale are attributed based on a semi-quantitative analysis of global atrophy, determined by the prominence of the ventricular spaces in detriment of the encephalic parenchyma and classified as follows: absent (0), slight (1) or severe (2). Hence, the final score ranged from 0 to 16, with higher scores indicating greater involvement.

**Statistical analyses.** An overall descriptive analysis of patients' DRS performance was first carried out and the group exhibiting impairmentson the DRS was then compared with the unimpaired group. The Mann-Whitney test was employed for continuous variables and Fisher's test for categorical variables. In order to investigate associations of categorical variables with global cognitive impairment, odds ratio (OR) values were calculated. Cognitive and motor performances were also compared according to specific topographies of MRI hyperintensities using the Mann-Whitney test. The associations found on the OR analysis were confirmed or otherwise bylogistic regression models, each with two variables. The first model was adjusted for education, the second for motor scale scores, while the third model was adjusted for total scores on the MRI scale. The p-value used for statistical significance was set at <0.05.

## RESULTS

Impaired scores on the DRS were observed in nine out of the 20 patients evaluated, with a median of 123 points for the impaired group and 140 points for the group with no impairment. Comparison between the clinical, cognitive and neuroimaging features of the overall sample are depicted in Table 1, together with those of the two subgroups of patients.

**Table 1:** Clinical, cognitive and magnetic resonance imaging features.

	WD - TS	WD - GCI N=9	WD-WGCI N=11	P*
Age	30.05±7.26	30.11 ±6.55	30 ±8.08	0.941
Sex (male)	11	7	4	0.092
Education (years)	11.15 ±3.73	9.11 ±3.58	12.82 ±3.06	0.031
Motor Symptoms Scale	2.79 ±1.87	3.56 ±1.74	2.1 ±1.79	0.095
Duration of disease (years)	11.75 ±7.79	10.89 ±7.11	12.45 ±8.59±	0.824
Penicillamine use	14	8	6	0.157
MMSE	26.7 ±2.45	25.22 ±2.58	27.91 ±1.57	0.016
M2	9.15 ±0.67	8.78 ±0.667	9.45 ±0.52	0.046
M5	8.5 ±1.39	7.78 ±1.30	9.09 ±1.22	0.056
AF	17.35 ±5.77	13.89 ±5.46	20.18 ±4.44	0.016
Clock drawing	8 ±2.44	7.13 ±2.58	8.64 ±2.24	0.129
FAS	22.4 ±12.40	17.56 ±13.03	26.36 ±10.85	0.046
STROOP (errors)	4.45 ±4.82	6.67 ±6.14	2.64 ±2.46	0.112
FAB	12.95 ±2.85	11.11 ±2.66	14.45 ±2.06	0.007
CERAD naming test	14.2 ±1.54	13.44 ±2.06	14.82 ±0.405	0.112
Cubes (WAIS)	7.80 ±2.87	6.67 ±3.04	8.73 ±2.49	0.080
Hooper	61.10 ±7.75	61.89 ±9.54	60.45 ±6.34	1
WCST	2.25 ±1.37	1.89 ±1.16	2.55 ±1.50	0.456
Pfeffer scale	2.10 ±2.69	3.33 ±2.39	1.09 ±2.58	0.016
MRI Total Score	7.70 ±2.99	9.44 ±2.74	6.27 ±2.45	0.016
MRI hyperintense T2 signal + atrophy	3.55 ±2.32	5.0 ±2.23	2.36 ±1.69	0.016
MRI hyperintense T2 signal - putamen	13	6	7	1.00
MRI hyperintense T2 signal - globus pallidus	7	6	1	0.017
MRI hyperintense T2 signal -thalamus/subthalamus	3	3	0	0.074
MRI hyperintense T2 signal -mesencephalon	8	6	2	0.065
MRI hyperintense T2 signal - pons	9	5	4	0.653
MRI hyperintense T2 signal - cerebellum	5	3	2	0.617
MRI hyperintense T2 signal - subcortical white matter	8	3	5	0.362

MMSE: Mini-Mental State Examination; M2: memory (learning); M5: memory (delayed recall); AF: animal fluency; FAS: phonemic fluency; FAB: Frontal Assessment Battery; WCST: Wisconsin Card Sorting Test; WD: Wilson’s disease; WD-GCI: Wilson’s disease with global cognitive Impairment; WD-WGCI+ Wilson’s disease without global cognitive impairment.

* WD-GCI × WD WGCI.

The group with DRS total score deficits performed significantly worsein comparison to the other subgroup on the Initiative/Perseveration and Conceptualization subscales, with medians of 29 vs. 37 and 32 vs. 38, respectively. No statistical difference was found for the other subscales.

Patients with impaired total DRS scores had higher frequencies of hyperintensity signal in the globus pallidus and in the mesencephalon. This observation led to separate assessment of those individuals with hyperintensity signal changes in the globus pallidus and mesencephalon, and comparisons to patients with no signal change in these topographies. The patients with hyperintensity signal in these regions had higher scores on the MSS, displayed global cognitive deficits (MMSE and DRS), changes in executive function (verbal fluency – animals and FAS), as well as changes in the learning phase of the memory test (globus pallidus) (Table 2).

**Table 2:** Clinical, cognitive and total magnetic resonance imaging in patients with specific topographic MRI changes.

	Hyperintense T2 signal Globus Pallidus (7)	Without Hyperintense T2 signal Mesencephalon Globus Pallidus (13)	p	Hyperintense T2 signal Mesencephalon (8)	Without Hyperintense T2 signal Mesencephalon (12)	p
Age	28.29±6.02	31.0±7.90	0.438	27.5±7.17	31.75±7.09	0.181
Motor Symptoms Scale	4.14±1.21	2.0±1.75	0.013	4.38±1.40	1.64±1.2	0.001
Education (Years)	9.71±3.54	11.92±3.73	0.311	9.25±5.06	12.42±1.83	0.02
Duration of disease (years)	10.14±6.14	12.62±8.66	0.699	9.5±6.27	13.25±8.59	0.427
MMSE	25.57±2.50	27.31±2.28	0.115	25.13±2.64	27.75±1.71	0.025
Animal fluency	13.14±4.74	19.62±5.50	0.019	12.38±4.43	20.67±3.86	0.001
FAS	15.43±10.76	26.15±11.91	0.037	14.25±9.09	27.83±11.51	0.012
M2	8.57±0.53	9.46±0.51	0.011	8.75±0.70	9.42±0.51	0.057
M5	7.86±1.46	8.85±1.28	0.251	7.75±1.16	9±1.34	0.082
FAB	12.14±1.95	13.38±3,22	0.211	11.75±2.76	13.75±2.73	0.098
Stroop 3	5.14±4.37	4.08±5.18	0.438	6.88±6.12	2.83±3.04	0.082
CERAD	14.0±1.91	14.31±1.37	0.938	13.63±2.264	14.58±0.66	0.678
DRS	126.14±12.21	135.85±8.58	0.012	124.5±12.75	137.75±4.53	0.012
Cubes (WAIS)	8.14±3.02	7.62±2.90	1.0	6.38±2.77	8.75±2.66	0.135
Hooper	62.43±10.58	60.38±6.13	0.877	64.13±9.56	59.08±5.86	0.208
WCST	2.29±0.95	2.23±1.58	0.699	2.13±1.35	2.33±1.43	0.910
Total MRI	9.86±1.95	6.54±2.84	0.019	9.88±2.10	6.25±2.63	0.010
Hyperintense T2 signal MRI	4.29±1.60	1.77±1.73	0.008	5.75±1.48	2.08±1.44	<0.001
Pfeffer scale	3.29±2.36	1.46±2.72	0.008	3.88±2.74	0.98±1.97	0.012

MMSE: Mini-Mental State Examination; M2: memory (learning); M5: memory (delayed recall); AF: animal fluency; FAS: phonemic fluency; FAB: Frontal Assessment Battery; DRS: Dementia Rating Scale; WCST: Wisconsin Card Sorting Test.

The neuroimaging factors related to poor global cognitive performance were determined by calculating the OR for each feature. The presence of hyperintensity signal in the globus pallidus (significance) and in the mesencephalon (tendency) were associated with a worse global performance (Table 3). The former association remained after correcting for education, but significance was lost when correcting for neurological and MRI scores. The hyperintensity signal in the mesencephal on did not have a statistically significant association.

**Table 3:** Association between MRI changes and cognitive impairment.

Topography Hyperintense T2 Signal	OR (95% CI)	Model A^+^ ß (95% CI)	Model B^++^ ß (95% CI)	Model C^+++^ ß (95% CI)
Globus pallidus	5.38 (0.85-33.86)*	22.14 (1.19-410.30)*	13.473 (0.82-220.09)	9.06 (0.627-131.16)
Mesencephalon	3.00 (0.86-10.40)	5.04 (0.44-57.87)	5.38 (0.283-102.47)	3.11 (0.287-33.814)

* p<0.05;

+ corrected for years of education;

++ corrected for Motor Symptoms Scale scores;

+++ Correction for MRI total scores.

## DISCUSSION

We observed that 45% of our sample had low total score on the DRS (below the 10th percentile). This rate is slightly higher to that found by Medalia in 1988.3 When compared to the group with normal performance, there was no difference in relation to age or time of symptom onset. This suggests that after at least one year of follow-up and continuous treatment, the occurrence of cognitive decline is not related to the duration of symptoms.

The use of D-Penicillamine also did not differ between the two groups. There are no previous studies assessing the role of specific treatments on cognitive symptoms in WD. The use of D-Penicillamine was associated with an improvement in the MRI parameters compared to Zinc, but without impact on motor changes.^20^ In order assess the impact of better treatment and disease duration on the occurrence of cognitive symptoms, a prospective study would be required in which patients with no previous treatment and cognitive evaluation prior to the study were selected and retested after a one-year follow up.

The population with worse cognitive performance tended to have higher MSS scores. The intensity of motor symptoms is associated to the quantity of altered cognitive tests^7^ and can also influence performance on some tests.^3^ We used tests with lower motor performance demands and excluded patients with serious motor problems. The small number of patients in each group might have prevented statistical significance from being reached in this case.

The tendency for a greater number of men with cognitive impairment has not been reported in previous studies. Our finding is probably related to the fact that men were less educated than women in our sample (9.82 vs. 12.78 years, respectively; p=0.031). Indeed, education was the only sociodemographic difference between the two groups. We used education-adjusted cut-off scores for the DRS, nevertheless, the population with lower educational level showed greater impairmenton the test. Low educational level is related to a greater risk for dementia,^21^ probably due to a smaller cognitive reserve.^22^ In the present study, no difference was observed in neuroimaging scale score for the group with ≤11years of education compared to those with > 11 years of schooling on the full MRI scale (7.77 vs 7.57 p=0.877), or for hypersignal (2.77 vs 2.43 p=0.877) or hyperintensity signal plus atrophy (3.77 vs 3.14 p=0.757). Furthermore, no correlation was detected between the number of years of education and scores on the MRI full scale (r2: –0.303 p=0.194). These findings suggest that for the same number of neuroimaging changes, individuals with less education are more susceptible to worse cognitive performance.

Cognitive performance in the population with impairment on the DRS was lower, especially in the executive function tests. The subscales with lowest performance were Initiation/Perseveration and Conceptualization, both associated with executive functions. This observation is in agreement with a previousstudy.^23^ Changes in response time,^23^ inhibitory control,^7^ selective attention^24^ and working memory^23^ have been reported in patients with WD presenting neurological symptoms. The absence of difference on the Stroop test might have occurred because this is a common deficit in patients with WD,^7^ even in cases whose performance on other cognitive tests is normal.

Memory deficits may be seen in patients with WD, more on encoding than in the delayed recall,^3,6,7^ as was seen in our sample. The normal performances on tests related to praxis and visuospatial abilities confirm that these are not domains affected in patients with WD.^3,23^

The pattern of preferential involvement of the executive functions and memory (learning) suggests dysfunction in cortico-subcortical circuits of the frontal lobe. In 2002, Seniow et al. reported that patients with lesions restricted to the basal nuclei also showed cognitive changes.^6^ A previous study seeking to correlated cognitive performance with MRI changes suggested that putamen involvement might be associated with a higher number of changes in cognitive tests.^8^ In the study, only 12 patients were evaluated, three of which had normal MRI, thus limiting the study to nine patients and preventing the use of any statistical evaluation. For this reason, the results were only descriptive.

In our sample, all 20 patients presented changes on brain MRI.^7^ The putamen was the topography most frequently affected (sign changes), but without difference in frequency between the groups with and without cognitive impairment. When we compared the seven patients with no putamen changes to the remaining,^13^ no statistical differences were found for any of the tests except the MSS, suggesting that changes in this topography impact motor more than cognitive performance.

In our study we found that the topographic association between cognitive and T2 hyperintensity signal changes had a tendency to be associated with involvement of the mesencephalon and attained statistical significance in the globus pallidus. Patients with changes in these topographies had worse performance on executive function tests, such as fluency and learning memory, but also had more severe motor symptoms and changes on MRI. Correcting for these factors decreased statistical significance, which persisted only after the adjustmentfor educational level.

Few studies have evaluated the role of the globus pallidus in executive functions or in global cognitive performance. One study entailing deep brain stimulation (DBS) in patients with Parkinson's disease indicated that this would be a safer surgical site than the subthalamic nucleus for preventing cognitive dysfunctions.^25^ A study of patients with generalized dystonia observed cognitive worsening, mainly on digit span, errors on attention tests and worse recall in the Rey Auditory Verbal Learning Test, after placement DBS probes into the internal globus pallidus.^26^

More recently, observing pathological studies in patients with Huntington's disease that had a similar cognitive profile to our sample,^7^ a linear correlation was found between atrophy of the external globus pallidus and its most ventral part with global cognitive dysfunction in these patients.^27^ This result, taken together with our findings, suggests that the globus pallidus plays more than merely motor roles in the basal nuclei and that changes in this topography are associated with worse cognitive performance.

The small number of patients in our group might have limited the assessment of our data and prevented attainment of statistical significance on some tests. The fact that we were unable to define which parts of the globus pallidus and the mesencephalon were more involved also prevented a better correlation between cognitive performance and neuroimaging changes.

Despite these limitations, our study confirms that global cognitive impairments occur in a considerable proportion of patients with WD. These changes are associated, as previously reported, with severity of neurological impairments^7^ low educational level, severity of hypersignal changes on MRI, andthe occurrence of these MRI changes in the globus pallidus.
